# Cardiovascular Biomarkers in Association with Dietary Intake in a Longitudinal Study of Youth with Type 1 Diabetes

**DOI:** 10.3390/nu10101552

**Published:** 2018-10-19

**Authors:** Namrata Sanjeevi, Leah M. Lipsky, Tonja R. Nansel

**Affiliations:** Social and Behavioral Sciences Branch, Division of Intramural Population Health Research, Eunice Kennedy Shriver National Institute of Child Health and Human Development, Bethesda, MD 20817, USA; leah.lipsky@nih.gov (L.M.L.); nanselt@mail.nih.gov (T.R.N.)

**Keywords:** type 1 diabetes, cardiovascular disease, whole grains, glycemic control, serum lipids

## Abstract

Despite cardioprotective effects of a healthy diet in the general population, few studies have investigated this relationship in individuals with type 1 diabetes, who are at elevated risks of cardiovascular disease (CVD) due to hyperglycemia. The objective of this study was to examine the association of CVD biomarkers with overall diet quality, as measured by the Healthy Eating Index-2015 (HEI-2015), and its dietary components in youth with type 1 diabetes. Youth with type 1 diabetes (*n* = 136, 8–16.9 years) were enrolled in an 18-month behavioral nutrition intervention trial. Dietary intake from three-day diet records, CVD biomarkers (total cholesterol (TC), high-density lipoprotein cholesterol (HDL-C), low-density lipoprotein cholesterol (LDL-C); triglycerides (TG), C-reactive protein (CRP), 8-iso-prostaglandin-F2alpha (8-iso-PGF_2α_), systolic and diastolic blood pressure (SBP and DBP, respectively), and glycated hemoglobin (HbA1c) were assessed at baseline, 6, 12 and 18 months. Linear mixed-effects models estimated associations of dietary intake with CVD biomarkers, adjusting for HbA1c and other covariates. Separate models estimated associations of time-varying change in dietary intake with time-varying change in CVD biomarkers. HEI-2015 was not associated with CVD biomarkers, but whole grain intake was inversely associated with TC, HDL-C and DBP, and a greater increase in whole fruit intake was associated with lower DBP. Added sugar, saturated fat and polyunsaturated fat were positively related to serum TG, HDL-C, and DBP, respectively. Findings suggest that the intake of specific dietary components, including whole grains, whole fruits, added sugar and PUFA, may influence cardiometabolic health in youth with type 1 diabetes, independent of glycemic control.

## 1. Introduction

Cardiovascular disease (CVD) is the major cause of mortality and morbidity in patients with type 1 diabetes, whose risk is several-fold higher than the general population [1,2]. This increased risk starts early in life; 15–45% of youth with type 1 diabetes have at least two CVD risk factors, and subclinical CVD abnormalities are observed within the first decade of diagnosis [3]. While glycemic control is central to CVD prevention in type 1 diabetes [4], identifying additional risk modifiers would inform timely interventions.

Several studies in the general population have indicated associations of dietary intake with CVD indicators; however, few studies have examined these relationships in individuals with type 1 diabetes. Better cardiometabolic health in the general population is consistently associated with better overall diet quality [5,6], greater intake of whole plant foods [7,8,9,10,11,12,13,14,15,16,17], and lower intakes of added sugar [18,19], saturated fat [20,21] and sodium [22,23]. Associations of dietary intake with CVD risk in individuals with type 1 diabetes may differ from that of the general population due to the characteristic glucose fluctuations and hyperglycemia that trigger inflammatory responses, thereby increasing CVD risk [24,25]. However, only two studies have examined associations of overall diet quality with CVD risk factors in individuals with type 1 diabetes, finding an inverse association of overall diet quality with blood pressure in adults [26] and dyslipidemia in youth [27,28]. Furthermore, associations of CVD biomarkers with the intake of individual food groups and nutrients other than fiber [29,30,31], saturated and total fat [31] in adults, and sugar-sweetened beverages [32] in youth have not been examined. Although diet quality indices reflect overall dietary patterns, a composite score based on several dietary components may mask associations of individual food groups or nutrients with cardiovascular health. Understanding associations with individual dietary components also is critical for developing behavioral targets for reducing CVD risk in patients with type 1 diabetes. Additionally, although oxidative stress and inflammation are known CVD risk factors [24], only one study has investigated dietary associations with inflammation in patients with type 1 diabetes, suggesting an inverse relationship of fiber intake with inflammation. No study has examined associations of diet with oxidative stress. Thus, the objective of this research was to investigate relationships of CVD biomarkers (serum lipids, blood pressure, inflammation and oxidative stress) with overall diet quality, as measured by Healthy Eating Index-2015 (HEI-2015), and its dietary components in youth with type 1 diabetes followed prospectively for 18 months.

## 2. Materials and Methods

This is a secondary analysis of a randomized controlled trial of a family-based behavioral nutrition intervention conducted from 2010–2013 at a tertiary diabetes center in Boston, Massachusetts. Youth eligibility criteria included: Age 8.0 to 16.9 years, diagnosed diabetes duration of ≥1 year, daily insulin dose of ≥0.5 units per kilogram, HbA1c of 6.5% (48 mmol/mol)–10.0% (86 mmol/mol) at the most recent clinic visit, insulin pump use or ≥3 injections per day, ≥1 clinic visit in the past year, and able to communicate in English. Youth were excluded due to the daily use of premixed insulin, the transition to insulin pump therapy three months prior to study, the use of real-time continuous glucose monitoring three months prior to study, participation in another intervention study six months prior to study, the use of medications that significantly interfere with glucose metabolism, the presence of gastrointestinal disease, multiple food allergies, or significant mental illness. Of 622 eligible youth, 24% (*n* = 148) provided consent and 22% (*n* = 139) completed baseline measures. Data from one sibling each of 3 sibling pairs was excluded resulting in 136 youth, and 125 participants were retained through the study duration of 18 months.

Intervention details have been described previously [33]. The intervention aimed to increase the intake of whole plant foods (whole fruits, vegetables, whole grains, legumes, nuts, and seeds). The control group had an equal frequency of contact with the research staff, but did not receive any nutrition advice besides that included as part of regular type 1 diabetes care.

Youth and parents provided assent and written informed consent, respectively, at the time of enrollment. Written informed consent was obtained from youth turning 18 during the study. Study procedures were approved by institutional review boards of the participating institutions. Sample size was ascertained to detect meaningful differences in dietary intake and HbA1c between the treatment and control groups [33].

### 2.1. Measures

#### 2.1.1. CVD Biomarkers

Youth blood and urine samples obtained at baseline, 6, 12 and 18 months were stored at room temperature for 20–30 min after withdrawal, centrifuged for 15 min at ~3000 RPM at 4 °C, then aliquoted and frozen at −80 °C for later analysis. Serum concentrations of triglycerides (TG), total cholesterol (TC), high-density lipoprotein cholesterol (HDL-C) and low-density lipoprotein cholesterol (LDL-C) were assessed using enzyme linked immunosorbent assay (ELISA). Systolic and diastolic blood pressure (SBP and DBP) were abstracted from medical records at each visit. A high sensitivity enzyme-linked immunosorbent assay (Architect c8000, Abbott) with a lower detection limit of 0.2 mg/L was used to quantify CRP in the serum samples, and 8-iso-prostaglandin F_2α_ (8-iso-PGF_2α_), an indicator of oxidative stress, was determined from urine frozen at −80 °C using enzyme linked immunosorbent assay (ELISA).

#### 2.1.2. Diet Assessment

Families completed 3-day youth diet records at baseline and 3, 6, 9, 12, and 18 months based on instructions from research assistants on accurate measurement and reporting of food and beverage intake. Potion size estimation was facilitated by scales and measuring utensils. Families were asked to include detailed description of each food item, such as brand names and item labeling (e.g., low fat or 1% milk). The completed records were reviewed by research staff, and clarified for any missing information, as needed. A registered dietitian obtained two nonconsecutive 24-h dietary recalls for visits in which a family did not complete the diet record (1.7% of assessments).

Three-day diet records were analyzed using Nutrition Data System for Research 2012 (NDSR 2012; Nutrition Coordinating Center, University of Minnesota) software to obtain intake estimates of key dietary components of the Dietary Guidelines of Americans 2015 (DGA 2015), including total and whole fruits, total vegetables, greens and beans, whole and refined grains, dairy, total protein foods, seafood, nuts and seeds, sodium, percentage of energy from added sugars and saturated fat, and fatty acids. Intake of food groups and sodium per 1000 kcal were determined. The dietary subgroup, fatty acids, is represented as the ratio of the sum of poly- and mono-unsaturated fat (PUFA and MUFA, respectively) to saturated fat ((PUFA + MUFA): SFA). Overall diet quality was measured using the Healthy Eating Index-2015 (HEI-2015), an a priori index that measures in adherence to DGA 2015 recommendations. The score is comprised of twelve component scores, which are summed to obtain the total score that ranges from 0 to 100; a greater score indicates greater conformance to the DGA 2015.

#### 2.1.3. Clinical and Demographic Data

HbA1c was assessed at baseline, 3, 6, 9, 12 and 18 months using a laboratory assay standardized to the Diabetes Control and Complications Trial (reference range, 4–6%, (20–42 mmol/mol)). Tosoh (Tosoh Medics, South San Francisco, CA, USA) was used for initial HbA1c assays ensued by Roche Cobas Integra (Indianapolis, IN, USA). No clinically significant bias was detected between the Tosoh and Roche methods based on results from the two instruments on identical samples.

Date of diabetes diagnosis, insulin regimen, age, sex, height, weight, Tanner stage and use of cardiac medications (anticoagulants, ace inhibitors and statins) were abstracted from the medical records at each study visit. Tanner stage from the previous visit was used for missing visit data. Body mass index (BMI, kg/m^2^) was computed from height and weight measurements, and baseline diabetes duration was calculated based on date of diagnosis. Parents reported youth race/ethnicity at baseline. Items from the Behavioral Risk Factor Surveillance System assessed total moderate and vigorous physical activity at baseline, 6, 12 and 18 months [34].

### 2.2. Statistical Analysis

Differences in characteristics between treatment groups were analyzed using independent samples *t*-tests for continuous variables and Pearson’s chi-square tests for categorical variables. Serum TG and CRP were log-transformed to improve normality. Linear mixed-effects models estimated associations of time-varying continuous independent variables (HEI-2015, and intake per 1000 kcal of total fruits, whole fruits, total vegetables, dark green vegetables, beans, whole grains, dairy, total protein foods, seafood, nuts and seeds, refined grains and sodium, percentage of energy from added sugars and saturated fat, and fatty acids), with time-varying continuous dependent variables (TG, TC, LDL-C, HDL-C, CRP, 8-iso-PGF_2α_, SBP and DBP). Models included a random intercept representing the subject-specific baseline variation in the outcome. Covariates included baseline sex, treatment assignment, and diabetes duration, and time-varying age, body mass index, Tanner stage, insulin regimen, physical activity, use of cardiac medications, and HbA1c. Adjustments for multiple comparisons were not performed due to the exploratory nature of the secondary data analysis [35,36]. Since 42.6%, 85.5%, 76.7% and 54.7% of participants reported zero intake of dark green vegetables, beans, seafood, and nuts and seeds, respectively, these variables were dichotomized as having either zero or greater than zero intake. Additional models estimated associations of each of the dichotomized food group variable with CVD risk factors. Further, to enable within-subject analysis of the association of change in diet with change in CVD biomarkers, the change from baseline in each dietary intake variable and CVD biomarkers was calculated for each assessment period. Separate models estimated associations of time-varying change in dietary intake with time-varying change in CVD biomarkers. Models were controlled for baseline age, sex, treatment assignment, diabetes duration, Tanner stage, insulin regimen, HbA1c, physical activity, body mass index and use of cardiac medication for the respective assessment period. SPSS version 21 were used for all analyses.

## 3. Results

Baseline demographic and diet characteristics for study participants are shown in Table 1 and Table 2, respectively. At baseline, 19.4%, 11.2%, 28.4%, and 3.7% of participants were not within the recommended concentrations [37,38] for TG (<150 mg/dL), TC (<200 mg/dL), LDL-C (<100 mg/dL) and HDL-C (≥35 mg/dL), respectively.

Associations between dietary intake and serum lipids after adjustments of HbA1c and other covariates are indicated in Table 3. In the adjusted model, serum lipids were not associated with overall diet quality. However, whole grain intake was inversely associated with TC and HDL-C. Added sugar and saturated fat intakes were positively associated with TG and HDL-C, respectively. Other dietary components (including dark green vegetables, beans, seafood, and nuts and seeds) were not associated with serum lipids. Findings were unchanged when the food groups, dark green vegetables, beans, seafood, and nuts and seeds, were treated as categorical variables (Table S1).

Associations of dietary intake with inflammation, oxidative stress and blood pressure are shown in Table 4. These CVD biomarkers were not associated with overall diet quality; however, 8-iso-PGF_2α_ was inversely associated with refined grain intake, and lower DBP was related to greater whole grain intake and greater (PUFA + MUFA): SFA. To determine the specific fatty acids associated with DBP, we further examined the association of the intake of individual fatty acid with DBP. Although MUFA intake (% kcal) was not associated with DBP (β = 0.08, SE = 0.10, *p* = 0.38), PUFA intake (% kcal) was positively related to DBP (β = 0.23, SE = 0.09, *p* = 0.008). Dark green vegetables, beans, seafood and nuts and seeds were not associated with inflammation, oxidative stress and blood pressure (Table 4), and findings were unchanged when these food groups were treated as categorical variables (Table S2).

Associations between time-varying changes in dietary intake with time-varying changes in CVD biomarkers are indicated in Tables S3 and S4 Greater increase in whole grain intake was associated with a decrease in LDL-C and HDL-C. Increase in whole fruit intake was inversely associated with DBP, whereas increase in fatty acid ratio was positively associated with DBP. Increase in time-varying total vegetable, whole grain and added sugar intake was positively associated with TG, but increase in time-varying refined grain intake was inversely associated with TG and 8-iso-PGF_2α_. Further, increase in time-varying dairy intake was positively associated with SBP.

## 4. Discussion

In contrast to the general population [5,39], CVD biomarkers were not associated with greater adherence to DGA 2015, as measured by HEI, in this sample of youth with type 1 diabetes. However, greater intake of whole grains and whole fruits, and lower added sugar and PUFA were associated with more favorable CVD biomarkers. Much of the current study findings regarding the association of intake of specific dietary components with CVD biomarkers are comparable to the general population. However, CVD etiology in type 1 diabetes differs from that of the general population, as hyperglycemia is known to increase CVD risk. Findings suggest that these dietary components may be associated with CVD risk in persons with type 1 diabetes independent of glycemic control.

Several studies in the general population have indicated the protective role of whole grains in reducing CVD risk factors, including blood pressure [40] and TC [7,8,10]. In contrast, no study has examined this relationship in individuals with type 1 diabetes. Improved endothelial function due to whole grain consumption [41] could explain the association of greater intake with lower blood pressure in this sample. The inverse association of whole grain intake with TC, independent of HbA1c could be attributed to increased clearance or decreased biosynthesis of cholesterol [42]. This pathway also is consistent with the inverse relationship between whole grain consumption and HDL-C found in this study. Although this result is in contrast to previous research in the general population [8,10], it is comparable to another study finding that a whole grain diet significantly lowered HDL-C in overweight and obese adults [43]. The inverse association of whole grain intake with HDL-C is not considered atherogenic in this sample, given the negligible number of participants (3.7%) with low HDL-C (<35 mg/dL) at baseline. However, it is important to consider the impact of whole grain consumption on HDL-C in future CVD-related research in patients with type 1 diabetes. Associations of whole grain intake with cholesterol are further supported by within-subject analyses, where increase in whole grain intake was inversely related to LDL-C and HDL-C. Further, improved endothelial function could explain the inverse association of whole fruits with DBP, which is consistent to previous research in the general population [44]. However, other relationships of time-varying changes in whole grain, refined grain and dairy intake with that of CVD biomarkers are not supported by known biological mechanisms. Findings for within-subject analyses should be interpreted with caution, given the modest degree of individual change over time, resulting in increased chances of spurious findings.

The positive association of added sugar intake with serum TG is consistent with previous literature in the general population [18,19]. This result could be attributed to hepatic de novo lipogenesis induction by common sugars [45], resulting in increased serum TG, independent of glycemic control. This finding is also somewhat comparable to another study in youth with type 1 diabetes, whereby increased sugar-sweetened beverage intake was related to greater serum lipids [46]. The positive association of saturated fat intake with HDL-C is comparable to previous research [47], and may be explained by increased transport rates of HDL-C ester by dietary fat [47]. While additional studies are needed, these findings indicate some consistency in the relationship of food group and nutrient intake with serum lipids in individuals with type 1 diabetes as compared with the general population.

The inverse relationship of refined grain intake with oxidative stress contrasts previous research indicating the detrimental effects of refined grain intake on CVD biomarkers [9]. This may be attributed to the use of urinary 8-iso-PGF_2α_ as an oxidative stress marker, which is a less sensitive measure than its metabolite, 2,3-dinor-5,6-dihydro-15-F2t-isoprostane [48]. Future research using more sensitive markers of oxidative stress could better elucidate the association of refined grain intake with oxidative stress. Finally, greater DBP was associated with increased PUFA intake. Given PUFA includes omega-6 and omega-3 PUFA, a high intake could be obtained for an individual consuming excess omega-6 and minimal omega-3 PUFA. Although total omega-6 PUFA intake could not be obtained for the current study, stimulation of vasoconstriction by omega-6 PUFA-derived eicosanoids [49] could explain the relationship of PUFA intake with DBP reported in this study.

The study findings are subject to certain limitations. These findings are observational, precluding inferences on causality. The eligibility criteria for the primary study, recruitment from a single clinic, 22% recruitment rate and predominantly white sample limit generalizability of the results to all youth with type 1 diabetes. Since youth with poor glycemic control (most recent HbA1c prior to recruitment >10.0%) were not recruited, the study may have excluded patients most affected by adverse CVD biomarkers. The lack of adjustment for multiple comparisons could have increased the family-wise error rate. Nevertheless, the detected associations of diet with CVD biomarkers are supported by biological mechanisms. Although food records are among the most valid method of assessing dietary intake, they are susceptible to reporting and response biases [50]. However, this study is strengthened by the prospective design utilizing repeated measures of exposures and outcomes, assessment of several CVD risk factors, and adjustment of potentially important covariates in the analyses.

## 5. Conclusions

Nutrition recommendations for youth with type 1 diabetes include general healthful eating for reducing risk of complications, and there are limited disease-specific nutrition guidelines. However, characteristic hyperglycemia in individuals with type 1 diabetes may attenuate dietary influences on CVD biomarkers. The current study suggests that overall diet quality was not associated with CVD biomarkers in youth with type 1 diabetes. However, specific dietary components were associated with CVD biomarkers, independent of glycemic control. Additional studies are needed to corroborate if intake of greater whole grains, lower added sugar and PUFA may favorably influence CVD biomarkers in this population. Examining whether these dietary associations differ by glycemic control could inform efforts that promote cardiovascular health in patients with type 1 diabetes.

## Figures and Tables

**Table 1:** Baseline demographic characteristics of youth with type 1 diabetes overall and by treatment assignment.

	Overall (*N* = 136)	Treatment (*N* = 66)	Control (*N* = 70)	*p*-Value
Age, years	12.7 ± 2.6	12.5 ± 2.7	13.0 ± 2.5	0.27
Body mass index, kg/m^2^	21.3 ± 4.2	21.0 ± 4.1	21.6 ± 4.3	0.37
Weight status ^1^				
Underweight	1 (0.7)	1 (1.5)	0 (0)	0.55
Normal weight	91 (66.9)	42 (63.6)	49 (70.0)	
Overweight	28 (20.6)	17 (25.8)	11 (15.7)	
Obese	16 (11.8)	6 (9.1)	10 (14.3)	
HbA1c, (%, (mmol/mol))	8.1 ± 1.0 (65 ± 11)	8.1 ± 1.1 (65 ± 12)	8.1 ± 1.0 (65 ± 12)	0.95
Duration of diabetes, years	6.0 ± 3.1	5.6 ± 2.5	6.4 ± 3.6	0.15
Insulin dose, Units/day	49.8 ± 26.8	46.9 ± 23.9	52.7 ± 29.3	0.24
Tanner stage	2.5 ± 1.4	2.4 ± 1.4	2.6 ± 1.5	0.38
Youth race/ethnicity				
Non-Hispanic white	123 (90.4)	58 (87.9)	65 (92.9)	0.17
Non-Hispanic black	5 (3.7)	2 (3.0)	3 (4.3)	
Hispanic	7 (5.2)	6 (9.1)	1 (1.4)	
American Indian/Alaska Native	1 (0.7)	0 (0)	1 (1.4)	
TG (mg/dL)	111.1 ± 56.9	101.3 ± 42.4	120.2 ± 66.8	0.05
TC (mg/dL)	165.2 ± 27.9	162.7 ± 24.3	167.6 ± 30.8	0.32
HDL-C (mg/dL)	56.6 ± 13.6	56.5 ± 14.0	56.6 ± 13.3	0.98
LDL-C (mg/dL)	86.4 ± 24.0	85.7 ± 19.7	87.0 ± 27.5	0.75
CRP (mg/L)	1.1 ± 1.9	0.9 ± 1.4	1.4 ± 2.3	0.09
8-iso-PGF_2α_ (ng/mL)	1.6 ± 1.3	1.7 ± 1.5	1.4 ± 1.1	0.33
SBP (mm Hg)	108.7 ± 7.1	108.6 ± 7.8	108.8 ± 6.5	0.90
DBP (mm Hg)	66.4 ± 5.5	66.7 ± 5.9	66.2 ± 5.2	0.60

TG, triglycerides; TC, total cholesterol; HDL-C, high-density lipoprotein cholesterol; LDL-C, low-density lipoprotein cholesterol; CRP, C-reactive protein; 8-iso-PGF2, 8-iso-prostaglandin-F2-alpha; SBP, systolic blood pressure; DBP, diastolic blood pressure. Data are presented as mean ± SD or *n* (%). ^1^ Underweight = BMI%ile < 5; Normal weight = 5 ≤ BMI%ile < 85; Overweight = 85 ≤ BMI%ile < 95; Obese = 95 ≤ BMI%ile.

**Table 2:** Baseline dietary characteristics of youth with type 1 diabetes overall and by treatment assignment.

Dietary Components	Overall (*N* = 136)	Treatment (*N* = 66)	Control (*N* = 70)	*p*-Value
HEI-2015	46.05 ± 11.70	45.33 ± 12.44	46.73 ± 11.01	0.49
Total fruits ^1^	0.44 ± 0.40	0.45 ± 0.38	0.42 ± 0.43	0.70
Whole fruits ^1^	0.32 ± 0.37	0.32 ± 0.32	0.33 ± 0.41	0.90
Total vegetables ^1^	0.49 ± 0.35	0.49 ± 0.38	0.49 ± 0.31	0.99
Dark green vegetables ^1^	0.09 ± 0.12	0.07 ± 0.11	0.10 ± 0.12	0.09
Beans ^1^	0.03 ± 0.09	0.04 ± 0.12	0.02 ± 0.05	0.19
Whole grains ^1^	0.52 ± 0.53	0.47 ± 0.38	0.57 ± 0.64	0.26
Dairy ^1^	0.68 ± 0.33	0.68 ± 0.34	0.67 ± 0.33	0.85
Protein foods ^1^	2.54 ± 1.24	2.53 ± 1.27	2.55 ± 1.21	0.94
Seafood ^1^	0.16 ± 0.40	0.11 ± 0.29	0.20 ± 0.48	0.07
Nuts and seeds ^1^	0.34 ± 0.73	0.33 ± 0.89	0.34 ± 0.54	0.93
Refined grains ^1^	3.44 ± 1.09	3.58 ± 1.04	3.30 ± 1.13	0.13
Sodium ^2^	1.69 ± 0.33	1.69 ± 0.31	1.68 ± 0.34	0.85
Added sugars, % kcal	11.97 ± 5.16	11.39 ± 4.47	12.53 ± 5.71	0.20
Saturated fat, % kcal	12.39 ± 2.75	12.37 ± 2.68	12.41 ± 2.83	0.93
Fatty acid ^3^	1.69 ± 0.49	1.72 ± 0.52	1.65 ± 0.46	0.42

^1^ Expressed as total number of cup- or ounce-equivalents per 1000 kcal; ^2^ expressed as grams per 1000 kcal; ^3^ calculated as (polyunsaturated fat + monounsaturated fat)/saturated fat.

**Table 3:** Association of diet quality and food group and nutrient intake with serum lipids in youth with type 1 diabetes ^1^.

Dietary Components	TG ^2^ (mg/dL)		TC (mg/dL)		HDL-C (mg/dL)		LDL-C (mg/dL)	
	β ± SE	*p*	β ± SE	*p*	β ± SE	*p*	β ± SE	*p*
HEI-2015	0.0003 ± 0.001	0.77	−0.16 ± 0.10	0.11	−0.07 ± 0.05	0.16	−0.12 ± 0.09	0.19
Total fruits ^3^	0.01 ± 0.03	0.78	1.31 ± 3.21	0.68	0.06 ± 1.49	0.97	1.07 ± 3.00	0.72
Whole fruits ^3^	0.01 ± 0.04	0.70	3.22 ± 3.96	0.42	0.67 ± 1.86	0.72	1.59 ± 3.68	0.67
Total vegetables ^3^	0.03 ± 0.03	0.28	2.44 ± 2.83	0.39	0.05 ± 1.36	0.97	−2.74 ± 2.66	0.30
Dark green vegetables ^3^	−0.02 ± 0.06	0.75	−9.18 ± 6.31	0.15	−2.63 ± 2.85	0.36	−8.50 ± 5.89	0.15
Beans ^3^	−0.14 ± 0.10	0.18	−17.17 ± 10.77	0.11	−2.81 ± 5.06	0.58	−5.16 ± 10.22	0.61
Whole grains ^3^	0.01 ± 0.02	0.61	−4.60 ± 2.05	0.03	−1.98 ± 0.99	0.046	−3.42 ± 1.92	0.08
Dairy ^3^	0.01 ± 0.03	0.79	−1.97 ± 3.78	0.60	−0.64 ± 1.81	0.72	−1.61 ± 3.44	0.64
Protein foods ^3^	−0.01 ± 0.01	0.30	0.05 ± 0.88	0.95	0.25 ± 0.41	0.55	0.39 ± 0.83	0.63
Seafood ^3^	0.01 ± 0.03	0.61	0.09 ± 2.66	0.97	−0.90 ± 1.27	0.48	1.77 ± 2.47	0.47
Nuts and seeds ^3^	0.004 ± 0.02	0.84	0.15 ± 1.74	0.93	0.12 ± 0.85	0.89	0.06 ± 1.64	0.97
Refined grains ^3^	−0.02 ± 0.01	0.08	0.54 ± 1.05	0.61	0.31 ± 0.51	0.54	1.72 ± 0.98	0.08
Sodium ^4^	−0.05 ± 0.03	0.10	2.13 ± 3.33	0.52	0.21 ± 1.52	0.89	5.38 ± 3.10	0.08
Added sugars, % kcal	0.004 ± 0.002	0.04	0.22 ± 0.21	0.30	−0.10 ± 0.10	0.32	0.09 ± 0.20	0.64
Saturated fat, % kcal	−0.002 ± 0.003	0.61	0.35 ± 0.37	0.35	0.34 ± 0.17	0.04	0.19 ± 0.35	0.58
Fatty acid ^5^	−0.002 ± 0.02	0.90	−0.50 ± 2.10	0.81	−0.74 ± 0.96	0.45	−1.12 ± 1.93	0.56

TG, triglycerides; TC, total cholesterol; HDL-C, high-density lipoprotein cholesterol; LDL-C low-density lipoprotein cholesterol; HEI-2015, healthy eating index-2015. ^1^ Models were controlled for baseline treatment assignment, sex, diabetes duration, and time-varying age, body mass index, Tanner stage, insulin regimen, physical activity, use of cardiac medication, and glycemic control (HbA1c); ^2^ log-transformed to meet the normality assumption; ^3^ expressed as total number of cup- or ounce-equivalents per 1000 kcal; ^4^ expressed as grams per 1000 kcal; ^5^ calculated as (polyunsaturated fat + monounsaturated fat)/saturated fat.

**Table 4:** Association of diet quality and food group and nutrient intake with inflammation, oxidative stress and blood pressure in youth with type 1 diabetes ^1^.

Dietary Components	CRP ^2^ (mg/L)		8-iso-PGF_2α_ (ng/mL)		SBP (mmHg)		DBP (mmHg)	
	β ± SE	*p*	β ± SE	*p*	β ± SE	*p*	β ± SE	*p*
HEI-2015	0.0002 ± 0.002	0.90	0.002 ± 0.007	0.77	0.01 ± 0.02	0.59	0.01 ± 0.02	0.53
Total fruits ^3^	0.04 ± 0.06	0.46	−0.23 ± 0.22	0.31	−0.25 ± 0.71	0.73	0.10 ± 0.66	0.88
Whole fruits ^3^	0.07 ± 0.08	0.39	−0.17 ± 0.28	0.54	−0.73 ± 0.86	0.40	−0.11 ± 0.88	0.90
Total vegetables ^3^	−0.02 ± 0.05	0.67	0.06 ± 0.21	0.77	−0.02 ± 0.69	0.97	0.69 ± 0.63	0.28
Dark green vegetables ^3^	−0.04 ± 0.12	0.72	−0.42 ± 0.50	0.40	−0.52 ± 1.46	0.72	−1.10 ± 1.38	0.42
Beans ^3^	−0.30 ± 0.21	0.15	0.85 ± 1.05	0.42	−0.64 ± 2.67	0.81	−0.93 ± 2.43	0.70
Whole grains ^3^	0.04 ± 0.04	0.26	−0.09 ± 0.16	0.60	0.03 ± 0.47	0.96	−0.98 ± 0.46	0.04
Dairy ^3^	−0.004 ± 0.07	0.95	−0.47 ± 0.27	0.08	0.89 ± 0.81	0.27	−0.99 ± 0.73	0.18
Protein foods ^3^	−0.01 ± 0.02	0.55	0.09 ± 0.07	0.22	−0.11 ± 0.20	0.59	0.20 ± 0.17	0.23
Seafood ^3^	0.02 ± 0.05	0.65	−0.09 ± 0.21	0.69	−0.18 ± 0.63	0.78	0.78 ± 0.61	0.20
Nuts and seeds ^3^	0.02 ± 0.04	0.61	0.25 ± 0.14	0.08	0.29 ± 0.45	0.53	0.21 ± 0.43	0.62
Refined grains ^3^	−0.02 ± 0.02	0.42	−0.18 ± 0.08	0.04	0.05 ± 0.25	0.85	0.04 ± 0.23	0.86
Sodium ^4^	0.02 ± 0.06	0.79	−0.42 ± 0.27	0.12	−0.53 ± 0.78	0.50	0.19 ± 0.78	0.80
Added sugars, % kcal	−0.00003 ± 0.004	0.99	0.01 ± 0.02	0.58	−0.04 ± 0.05	0.34	−0.01 ± 0.04	0.89
Saturated fat, % kcal	0.01 ± 0.007	0.43	0.02 ± 0.03	0.57	0.01 ± 0.08	0.87	−0.12 ± 0.08	0.13
Fatty acid ^5^	0.02 ± 0.04	0.59	0.19 ± 0.15	0.20	0.19 ± 0.45	0.67	0.98 ± 0.42	0.02

CRP, C-reactive protein; 8-iso-PGF_2α_, 8-iso-prostaglandin F_2α_; SBP, systolic blood pressure; DBP, diastolic blood pressure; HEI-2015, healthy eating index-2015. ^1^ Models were controlled for baseline treatment assignment, sex, diabetes duration, time-varying age, body mass index, Tanner stage, insulin regimen, physical activity, use of cardiac medication and glycemic control (HbA1c); ^2^ log-transformed to meet the normality assumption; ^3^ expressed as total number of cup- or ounce-equivalents per 1000 kcal; ^4^ expressed as grams per 1000 kcal; ^5^ calculated as (polyunsaturated fat + monounsaturated fat)/saturated fat.

## Supplementary Materials

The following are available online at http://www.mdpi.com/2072-6643/10/10/1552/s1.
