# Supplementary material for: Cardiovascular Biomarkers in Association with Dietary Intake in a Longitudinal Study of Youth with Type 1 Diabetes
Source: Nutrients. 2018 Oct 19;10(10):1552. doi: 10.3390/nu10101552 (PMC6213457; doi:10.3390/nu10101552)
Supplement: Supplementary file 1 [file nutrients-10-01552-s001.pdf]

Table S1. Association of food group intake (dichotomized as any vs none) with serum lipids in youth with type 1 diabetes [1].

Dietary Components	TG [2] (mg/dL)		TC (mg/dL)		HDL-C (mg/dL)		LDL-C (mg/dL)	
	β ± SE	*p*	β ± SE	*p*	β ± SE	*p*	β ± SE	*p*
Dark green vegetables [3]	-0.01±0.02	0.62	-1.42±2.18	0.52	0.57±1.02	0.58	-1.01±2.04	0.62
Beans [3]	-0.04±0.03	0.18	-2.39±2.79	0.39	-0.30±1.28	0.81	0.33±2.57	0.90
Seafood [3]	-0.01±0.02	0.58	0.32±2.56	0.90	0.20±1.21	0.87	1.94±2.37	0.41
Nuts and seeds [3]	0.02±0.02	0.30	-0.45±2.26	0.84	-0.56±1.03	0.59	-0.76±2.10	0.72

TG, triglycerides; TC, total cholesterol; HDL-C, high-density lipoprotein cholesterol; LDL-C low-density lipoprotein cholesterol. [1] Models were controlled for baseline treatment assignment, sex, diabetes duration, and time-varying age, body mass index, Tanner stage, insulin regimen, physical activity, use of cardiac medication, and glycemic control (HbA1c); [2] log-transformed to meet the normality assumption; [3] expressed as categorical variable.

Table S2. Association of food group intake (dichotomized as any vs none) with inflammation, oxidative stress and blood pressure in youth with type 1 diabetes [1].

Dietary Components	CRP [2] (mg/L)		8-iso-$PGF_{2\alpha}$ (ng/mL)		SBP (mmHg)		DBP (mmHg)	
	β ± SE	*p*	β ± SE	*p*	β ± SE	*p*	β ± SE	*p*
Dark green vegetables [3]	-0.0004±0.04	0.99	-0.03±0.17	0.86	0.26±0.50	0.60	0.01±0.51	0.98
Beans [3]	-0.08±0.05	0.14	0.33±0.22	0.13	0.62±0.66	0.35	-1.01±0.63	0.11
Seafood [3]	0.05±0.05	0.32	-0.03±0.19	0.88	-0.13±0.58	0.82	0.59±0.55	0.28
Nuts and seeds [3]	-0.01±0.04	0.83	-0.10±0.17	0.54	0.26±0.50	0.61	-0.35±0.47	0.46

CRP, C-reactive protein; 8-iso- $PGF_{2\alpha}$, 8-iso-prostaglandin F2α; SBP, systolic blood pressure; DBP, diastolic blood pressure. [1] Models were controlled for baseline treatment assignment, sex, diabetes duration, and time-varying age, body mass index, Tanner stage, insulin regimen, physical activity, use of cardiac medication, and glycemic control (HbA1c); [2] log-transformed to meet the normality assumption; [3] expressed as categorical variable.

Table S3. Association of time-varying change in diet quality, and food group and nutrient intake with time-varying change in serum lipids in youth with type 1 diabetes[1].

Dietary components	TG (mg/dl)		TC (mg/dL)		HDL-C (mg/dL)		LDL-C (mg/dL)	
	β ± SE	*p*	β ± SE	*p*	β ± SE	*p*	β ± SE	*p*
HEI-2015	–0.01 ± 0.31	0.98	–0.04 ± 0.11	0.70	–0.03 ± 0.05	0.50	–0.09 ± 0.10	0.37
Total fruits [2]	–0.69 ± 10.14	0.95	4.06 ± 3.46	0.24	0.23 ± 1.50	0.88	2.90 ± 3.17	0.36
Whole fruits [2]	1.07 ± 12.26	0.93	5.42 ± 4.19	0.20	0.43 ± 1.92	0.82	3.42 ± 3.85	0.38
Total vegetables [2]	21.48 ± 9.35	0.02	3.68 ± 3.06	0.23	–0.18 ± 1.39	0.90	–3.55 ± 2.79	0.20
Dark green vegetables [2]	1.11 ± 18.68	0.95	–9.49 ± 6.65	0.16	–2.97 ± 2.83	0.30	–11.22 ± 6.05	0.07
Beans [2]	–39.86 ± 35.84	0.27	–11.27 ± 11.36	0.32	0.11 ± 5.28	0.98	–3.90 ± 10.00	0.70
Whole grains [2]	15.28 ± 6.24	0.02	–4.06 ± 2.14	0.06	–2.12 ± 0.98	0.03	–4.28 ± 1.96	0.03
Dairy [2]	12.57 ± 11.90	0.29	–2.80 ± 3.95	0.48	–2.57 ± 1.88	0.17	–0.97 ± 3.59	0.79
Protein foods [2]	–4.95 ± 2.62	0.06	0.67 ± 0.89	0.45	0.59 ± 0.42	0.16	1.27 ± 0.80	0.11
Seafood [2]	5.57 ± 7.59	0.46	1.64 ± 2.51	0.52	–0.36 ± 1.27	0.78	1.57 ± 2.29	0.49
Nuts and seeds [2]	–5.86 ± 5.52	0.29	–0.30 ± 1.80	0.87	0.79 ± 0.93	0.40	–0.97 ± 1.66	0.56
Refined grains [2]	–8.39 ± 3.16	0.008	0.41 ± 1.07	0.70	0.43 ± 0.52	0.41	1.65 ± 0.98	0.10
Sodium [3]	–0.02 ± 0.01	0.07	0.003 ± 0.003	0.41	0.0002 ± 0.002	0.90	0.01 ± 0.003	0.05
Added sugars, % kcal	1.57 ± 0.65	0.02	–0.04 ± 0.22	0.85	–0.16 ± 0.10	0.11	–0.11 ± 0.20	0.59
Saturated fat, % kcal	–0.18 ± 1.07	0.86	0.15 ± 0.37	0.69	0.20 ± 0.17	0.23	0.18 ± 0.34	0.60
Fatty acid [4]	–2.69 ± 5.87	0.65	1.06 ± 2.02	0.60	0.55 ± 0.93	0.55	–0.73 ± 1.85	0.69

TG, triglycerides; TC, total cholesterol; HDL-C, high-density lipoprotein cholesterol; LDL-C low-density lipoprotein cholesterol; HEI-2015, healthy eating index-2015;
[1] models were controlled for baseline treatment assignment, sex, diabetes duration, age, Tanner stage, and insulin regimen, and body mass index, physical activity, use of cardiac medication, and glycemic control (HbA1c) for the respective assessment period; [2] expressed as total number of cup- or ounce-equivalents per 1000 kcal; [3] expressed as grams per 1000 kcal; [4] calculated as (polyunsaturated fat + monounsaturated fat)/saturated fat.

Table S4. Association of time-varying change in diet quality, and food group and nutrient intake with time-varying change in inflammation, oxidative stress and blood pressure in youth with type 1 diabetes [1].

Dietary components	CRP (mg/L)		8-iso-PGF$_{2\alpha}$ (ng/mL)		SBP (mmHg)		DBP (mmHg)	
	β ± SE	*p*	β ± SE	*p*	β ± SE	*p*	β ± SE	*p*
HEI-2015	–0.001 ± 0.02	0.98	0.01 ± 0.01	0.21	0.01 ± 0.03	0.71	0.02 ± 0.03	0.49
Total fruits [2]	0.76 ± 0.62	0.22	0.08 ± 0.26	0.76	–0.95 ± 0.84	0.26	–0.98 ± 0.80	0.22
Whole fruits [2]	1.30 ± 0.77	0.09	–0.02 ± 0.42	0.97	–2.00 ± 1.02	0.05	–2.04 ± 1.00	0.04
Total vegetables [2]	–0.34 ± 0.56	0.55	0.35 ± 0.25	0.17	0.07 ± 0.84	0.93	1.08 ± 0.81	0.19
Dark green vegetables [2]	–0.95 ± 1.14	0.41	0.06 ± 0.49	0.91	0.70 ± 1.64	0.67	–0.42 ± 1.59	0.79
Beans [2]	–1.46 ± 2.24	0.51	0.51 ± 1.17	0.66	4.86 ± 3.13	0.12	2.26 ± 2.98	0.45
Whole grains [2]	0.01 ± 0.39	0.97	0.12 ± 0.17	0.47	–0.02 ± 0.55	0.98	–0.20 ± 0.54	0.71
Dairy [2]	–0.32 ± 0.75	0.67	–0.44 ± 0.43	0.31	2.33 ± 1.02	0.02	–0.58 ± 1.02	0.58
Protein foods [2]	–0.07 ± 0.17	0.68	0.06 ± 0.10	0.57	–0.16 ± 0.24	0.50	0.24 ± 0.23	0.30
Seafood [2]	–0.94 ± 0.50	0.06	0.10 ± 0.21	0.63	–0.76 ± 0.66	0.25	1.06 ± 0.66	0.11
Nuts and seeds [2]	0.03 ± 0.34	0.94	0.07 ± 0.16	0.69	0.13 ± 0.49	0.79	0.06 ± 0.49	0.90
Refined grains [2]	–0.17 ± 0.20	0.40	–0.21 ± 0.09	0.02	–0.06 ± 0.28	0.82	–0.10 ± 0.27	0.70
Sodium [3]	–0.0004 ± 0.001	0.52	–0.0004 ± 0.0003	0.19	–0.001 ± 0.001	0.41	0.001 ± 0.001	0.32
Added sugars, % kcal	–0.02 ± 0.04	0.66	0.02 ± 0.02	0.30	–0.01 ± 0.06	0.92	–0.07 ± 0.06	0.21
Saturated fat, % kcal	0.04 ± 0.07	0.54	–0.01 ± 0.03	0.71	0.01 ± 0.09	0.96	–0.08 ± 0.09	0.40
Fatty acid [4]	0.23 ± 0.37	0.54	0.05 ± 0.15	0.74	–0.07 ± 0.52	0.89	1.06 ± 0.51	0.04

CRP, C-reactive protein; 8-iso-prostaglandin F2α, 8-iso- PGF$_{2\alpha}$; SBP, systolic blood pressure; DBP, diastolic blood pressure; HEI-2015, healthy eating index-2015;
[1] Models were controlled for baseline treatment assignment, sex, diabetes duration, age, Tanner stage, and insulin regimen, and body mass index, physical activity, use of cardiac medication, and glycemic control (HbA1c) for the respective assessment period; [2] expressed as total number of cup- or ounce-equivalents per 1000 kcal; [3] expressed as grams per 1000 kcal; [4] calculated as (polyunsaturated fat + monounsaturated fat)/saturated fat.
